# Preliminary Screening of Antioxidant and Antibacterial Activities and Establishment of an Efficient Callus Induction in* Curculigo latifolia* Dryand (Lemba)

**DOI:** 10.1155/2016/6429652

**Published:** 2016-05-19

**Authors:** Reza Farzinebrahimi, Rosna Mat Taha, Kamaludin A. Rashid, Bakrudeen Ali Ahmed, Mahmoud Danaee, Shahril Efzueni Rozali

**Affiliations:** ^1^Institute of Biological Sciences (IBS), Faculty of Science, University of Malaya, 50603 Kuala Lumpur, Malaysia; ^2^Biology Division, Center for Foundation Studies in Science, University of Malaya, 50603 Kuala Lumpur, Malaysia; ^3^Academic Development Centre (AdeC), Wisma R&D, University of Malaya, 59990 Kuala Lumpur, Malaysia

## Abstract

Leaf, seed, and tuber explants of* C. latifolia* were inoculated on MS medium supplemented with various concentrations of BAP and IBA, alone or in combinations, to achieve* in vitro* plant regeneration. Subsequently, antioxidant and antibacterial activities were determined from* in vitro* and* in vivo* plant developed. No response was observed from seed culture on MS media with various concentrations of PGRs. The highest percentage of callus was observed on tuber explants (94%) and leaf explants (89%) when cultured on MS media supplemented with IBA in combination with BAP. A maximum of 88% shoots per tuber explant, with a mean number of shoots (8.8 ± 1.0), were obtained on MS medium supplemented with combinations of BAP and IBA (2.5 mg L^−1^). The best root induction (92%) and mean number (7.6 ± 0.5) from tuber explants were recorded on 2.5 mg L^−1^ IBA alone supplemented to MS medium. The higher antioxidant content (80%) was observed from* in vivo *tuber. However, tuber part from the intact plant showed higher inhibition zone in antibacterial activity compared to other* in vitro* and* in vivo* tested parts.

## 1. Introduction

There are more than 20 species in the family Hypoxidaceae where* Curculigo* and* Hypoxis* are the two main genera of this family. The genus* Curculigo* is distributed in the tropical region of Africa and Asia rainforest's, particularly Malaysia and Singapore [1]. The four main species of this genus include* C. latifolia*,* C. capitulations*,* C. racemes,* and* C. orchioides* [2].

*Curculigo latifolia* Dryand, Lemba in Malaysia, is one of the important traditional Chinese medicinal plants. These species are propagated by rhizomes [3] and may be found abundantly in highland areas (with 1500–2000-meter altitude) and normally on slopes and forests. The leaf fibres can be used for making the fishing net, rope, and twines in Borneo and Malaysia. The leaves and flowers can treat a high fever. Flower and root concoctions are used to ease stomach-ache and frequent urination [4]. However, the rhizome dressing is applied externally to cure the cut and wounds [5]. In addition, it is reported to have an inhibitory effect on hepatitis B virus by rhizome extract [6]. Yamashita et al. [7] revealed two unique sweet proteins in the fruit such as Curculin and Neoculin that exhibit both sweet-tasting and taste-modifying characteristics at the same time. These proteins have been proven 500 times sweeter than sucrose by weight [8, 9] which could be employed as a low-calorie sweetener for diabetes or obesity [2]. To date, more than hundred compounds of secondary metabolites (such as phenols and phenolic glycosides), two polysaccharides (COPb-1 and COPf-1), and three proteins (Curculin, Neoculin, and *β*-amylase) have been known and extracted from this species. However, the phenols and phenolic glycosides from this plant are categorised as benzyl benzoate glycosides, followed by phenol glycosides and simple phenol. The 20 unique saponins are cycloartane triterpenoids and could be found in this species [10].

Grzegorczyk et al. [11] reported the* in vitro* acetone extract of* Salvia officinalis* exhibited antioxidant properties. However, many authors stated that* in vitro* and* in vivo* plant extracts showed antioxidant activities [12–15].

The increase in demands of this plant for commercial use requires an alternative rate of proliferation. During these years,* in vitro* technique is being widely applied to produce identical quality and disease-free plants [16]. There is no evidence on propagation of* C. latifolia* from seeds, but we reported that it is being propagated through rhizome [3]. However, due to high medicinal, industrial, and unique value of its compounds and low productivity and failed attempts for plantation of this species by conventional methods in nurseries, propagation of this plant by* in vitro* or tissue culture technique is mandatory.

An* in vitro* method for propagation of the species* C. orchioides* was established as a rare and endangered species in India [17–20]. However, some success of* in vitro* culture of this species has been reported [21].

The present study is advancement over the earlier protocol, because it describes the PGRs regulation,* in vitro* plant regeneration, and the role of regeneration plantlets in antioxidant and antibacterial effects to compare with* in vivo C. latifolia* and the subsequent transplantation of the plantlets to natural environmental conditions.

## 2. Plant Materials and Sterilization

The fresh mature fruits of* C. latifolia*, grown at Genting Highlands, Malaysia, were collected in the middle of September 2014. The tiny black seeds were obtained from dry fruits. Some botanists identified the plant materials and some pots were deposited at The University of Malaya (Green House, Institute Biological Sciences). The seed explants of* C. latifolia* were surface sterilized according to Taha [22] with some modifications.

The seeds were treated with 70%, 50%, 20%, and 10% (v/v) commercial bleach (Clorox) for 1 min at each concentration. The treated seeds were submerged in 70% (v/v) ethanol and finally by 3 times rinsing with sterile distilled water. Two drops of Tween-20 were also added during the treatment with 100% (v/v) Clorox to facilitate the sterilization process and reduce surface tensions.

Leaves and tubers were collected from (5-6 months old) seedlings, which were grown in Genting Highlands, Malaysia. Both types of explants were washed thoroughly under running tap water for 20 min. The leaves were soaked with commercial bleach or Clorox (70%) for 3 min under laminar flow and were rinsed 2 times with sterile distilled water. Treated leaves were submerged in 70% (v/v) ethanol and finally were rinsed 5 times with sterile distilled water.

The tubers were dipped in 30% citric acid to remove phenolic content and were immersed in 75% Clorox for 10 min. The tubers were sterilized by ethanol 100% (v/v) containing 0.1% (V/V) Tween 20 for 5 min and finally were rinsed five times with sterile distilled water.

## 3. Medium, Plant Growth Regulators (PGRs), and Callus Induction

For all treatments, MS basal medium [23] containing 3% sucrose was solidified with 2.5 g L^−1^ Gelrite (Duchefa brand, Netherland) and supplemented with various concentrations of IBA either alone or in combination with BAP (0.5–4 mg L^−1^).

All the media were adjusted to pH of 5.7 ± 0.1 with 0.1 N KOH prior to autoclaving at 121°C for 20 min. The media were dispensed into 60 mL specimen presterilized containers under laminar flow in aseptic condition.

The leaves were further cut into approximately 0.5 cm^2^ pieces, removing leaf ribs, and any other major leaf veins before being placed on the culture medium. The explants (3 per plate) were arranged horizontally and were pressed lightly into the surface of the culture medium.

Tubers (3 per plate) were cut into 2 cm^2^ and placed on the surface of the culture medium. In order to avoid the death of explants due to phenolic exudation, the tuber explants were subcultured three times every 3 days to the same media and PGRs.

The leaves and tuber explants were inoculated on MS basal media and supplemented either alone or in combination with IBA and BAP. The cultures were maintained at 25°C under a 16–8-hour photoperiod at a photon flux rate of 60 *μ*mol m^−2^ s^−1^ provided by cool daylight fluorescent lamps.

### 3.1. *In Vitro* Callus Growth

Callus cultures were optimized and measured for its biomass and secondary metabolic content. By applying various concentrations of PGRs, fresh and dry weights of the callus were determined at 8 weeks. At the end of the period, for all the treatments, each callus was harvested by careful separation from media using metal spatulas, and fresh and dry weight were promptly recorded.

### 3.2. Acclimatization

The regenerated plantlets were transplanted to 6 cm plastic pots filled with perlite and pit (3 : 1) and were kept in a controlled condition chamber with 80–90% relative humidity under a 16/8 h (light/dark) of photoperiod at a photon flux rate of 60 *μ*mol m^−2^ s^−1^ provided by cool daylight fluorescent for 8 weeks. The plantlets were transferred to greenhouse conditions after 8 weeks.

### 3.3. Plant Extraction

*In vivo* samples from young and healthy leaves and tuber (5-6 months old) were collected. The* in vitro* developed friable callus (without roots and shoots) from leaf and tuber explants was collected and both* in vivo* and* in vitro* samples were dried separately at room temperature for 4 days. To produce a fine homogenous powder, the samples were ground by electric blender. The 5 g of dried* in vivo* sample and* in vitro* regenerated callus of* C. latifolia* was extracted five times with ethanol [24]. The ethanol extract was centrifuged for 5 min at 5000 rpm. The supernatant was carefully pipetted into Eppendorf tubes. The plant extract (10 g L^−1^) was dissolved in phosphate buffered saline (PBS) and was kept at 4°C.

## 4. Antioxidant Activity

In order to study antioxidant properties, radical scavenging and superoxide dismutase assay were applied and the obtained results were compared.

### 4.1. Radical Scavenging Capacity Assay

DPPH^*∗*^ (2, 2-diphenyl-1-picrylhydrazyl) free radical scavenging capacity assay was achieved using the protocol described by Rafat et al. [15]. DPPH (950 *μ*L) at a concentration of 90 *μ*M was mixed with 50 *μ*L plant extract (10 g L^−1^), and the volume was adjusted to 4 mL using ethanol (95%). The solution was incubated for 120 min at room temperature in the dark condition. Scavenging of DPPH reduced the color of the solution and was measured using a spectrophotometer at 515 nm.

Comparison of the reduction of color in the examined samples with the blank (solution without plant extract) was used to measure the potential of scavenging capacity of the plant extracts using the following formula [15]:

(1) Radical  scavenging  capacity%=blank−sampleAblank×100.

### 4.2. Superoxide Dismutase Assay

Superoxide dismutase (SOD) determination kit (19160), ascorbic acid (A4544) from Sigma-Aldrich (St. Louis, Mo), and Tert-butylated hydroxytoluene (34750) from Fluka (Spain), were used for this part of the study. Plant extracts with a concentration of 10 g L^−1^ were added to 200 *μ*L of the kit working solution. The mixture, after gentle shaking, was incubated at 37°C for 20 min after adding 20 *μ*L of the kit enzyme working solution. The absorbance of the mixtures was measured at 450 nm using a microplate reader (BIO-RAD Model 550, USA) and the SOD activity was calculated based on the following equation [11]. Ascorbic acid (1 g L^−1^) and BHT or tert-butylated hydroxytoluene (1 g L^−1^) were employed as the positive controls in this study:

(2) Inhibition%SOD  activity=blank1−blank2−sampleA−blanksampleAblank1−blank2×100,

where blank_1_ = blank  of  mixture  working  solution + enzyme  working  solution + double  distilled  water, blank_2_ = blank  of  mixture  plant  extract + working  solution + dilution  buffer + double  distilled  water, blank_sample_A__ = blank  of  mixture  plant  extract + working  solution + dilution  buffer.

## 5. Antibacterial Activity Assay (Disk Diffusion Method)

The antibacterial potential of* C. latifolia* was investigated based on the paper disc diffusion method adopted from Farzinebrahimi et al. [12] with minor modification. Two gram-positive bacteria (*Staphylococcus aureus *and* Bacillus cereus*) and two gram-negative pathogenic bacteria (*Pseudomonas aeruginosa *and* Klebsiella *sp.) were obtained from Microbiology Division of Institute of Biological Sciences, University of Malaya, and maintained in a nutrient broth medium to produce a final concentration of 107 colony forming units (CFU) per mL. The test bacteria (0.1 mL) were streaked by sterile cotton swab on Mueller Hinton medium (MH) plates. Sterilized filter paper discs were soaked in extracts (10 g L^−1^) and then placed at the centre of test bacteria plates. The diameters of the inhibition zones were recorded after 24 hours of incubation at a temperature of 37°C overnight. Ampicillin (30 *μ*g) was applied as positive and negative controls, respectively. Sterile paper disks were put in samples; sterile distilled water and kanamycin antibiotic were as control for two hours.

## 6. Statistical Analysis

The experiments were conducted in a factorial based on randomized completely design with 4 blocks and 16 treatments. For all treatments, the mean and standard error were calculated. The data were analyzed by ANOVA followed by mean comparison using Duncan multiple range test (DMRT) [25]. Data were subjected to normality test using one sample Kolmogorov-Smirnov. All data analysis was done using SPSS ver. 21.

## 7. Results

### 7.1. Callus Induction

The seeds did not respond to MS media and different PGRs either alone or in combination after eight weeks of seed inoculation. It may be due to the hard seed coat, immature embryo, rudimentary embryo, and inhibitor substances. However, the callus initiation did not form without PGRs (plant growth regulators, control) in tuber and leaf explants.

The best callus induction from tuber explants was recorded in MS media with combinations of IBA and BAP (94%), whereas the same media supplemented with IBA and BAP alone produced 71% and 79% callus, respectively. This pattern was observed in callus formation from leaf explants, when combination of IBA and BAP produced higher percentage of callus (89%) as compared with IBA (82%) and BAP (83%).

**Figure 1:**
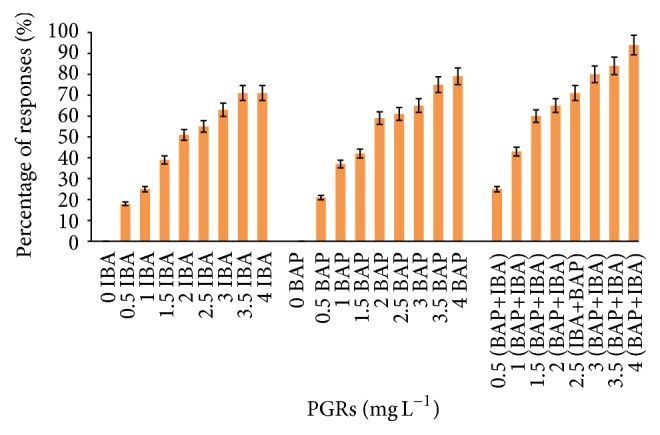
Callus formation from tuber explants of* C. latifolia* Dryand cultured on MS media supplemented with BAP alone or in combination with IBA (IBA+BAP) at various concentrations after 8 weeks of culture. No response was observed in IBA or BAP alone.

**Figure 2:**
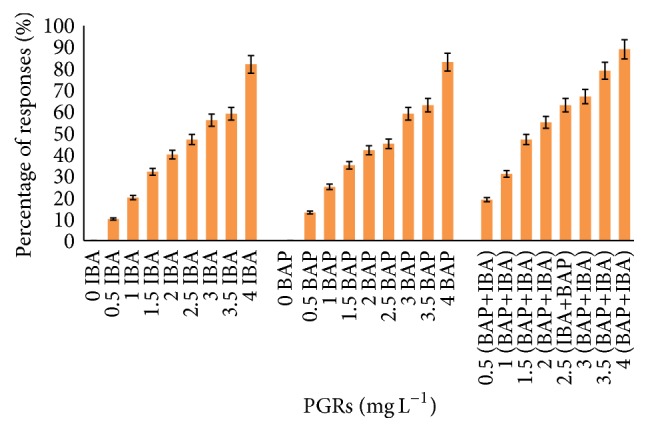
Callus formation from leaf explants of* C. latifolia* Dryand cultured on MS media supplemented with BAP alone or in combination with IBA (IBA+BAP) at various concentrations after 8 weeks of culture. No response was observed in IBA or BAP alone.

Among the various concentrations of applied PGRs in callus induction from tuber explants, IBA (3.5 and 4 mg L^−1^) and BAP (4.0 mg L^−1^) induced nature callus with a maximum of fresh and dry weight. However, the highest callus was formed in leaf explants when IBA and BAP (4.0 mg L^−1^) were applied alone or in combination (Figures 1 and 2).

### 7.2. Regeneration

The root and shoot formation were observed after 21 and 28 days of tuber inoculation and 16 and 21 days after leaf culture, respectively. The shoot and root formation were found in both tuber and leaf explants. However, the shoots formed in all concentrations of BAP alone or in combination with different concentrations of IBA with regard to the mean number of shoots and roots' elongation per explants. Control treatments involving no plant growth regulators, as well as those treatments that did not use IBA alone, produced no shoots at all in either the tuber or the leaf explants.

### 7.3. Root and Shoot Formation

#### 7.3.1. Tuber Explants

As illustrated in Table 1, the tuber explants inoculated in MS media supplemented with combinations of IBA and BAP (2.5 mg L^−1^) showed the highest number of shoots (8.8 ± 1.0). However, the best root numbers (7.6 ± 0.5) were formed in the same media added with IBA (2.5 mg L^−1^) alone after eight weeks of culture.

#### 7.3.2. Leaf Explants

The highest number of roots/explants was obtained from leaves (7.3 ± 0.1), when leaf explants were cultured on MS media supplemented with 2 mg L^−1^ IBA alone. However, combinations of BAP and IBA (2 mg L^−1^) produced 5.7 ± 0.8 shoots/explants (Table 2).

### 7.4. Root and Shoot Elongation

#### 7.4.1. Leaf Explants

**Figure 3:**
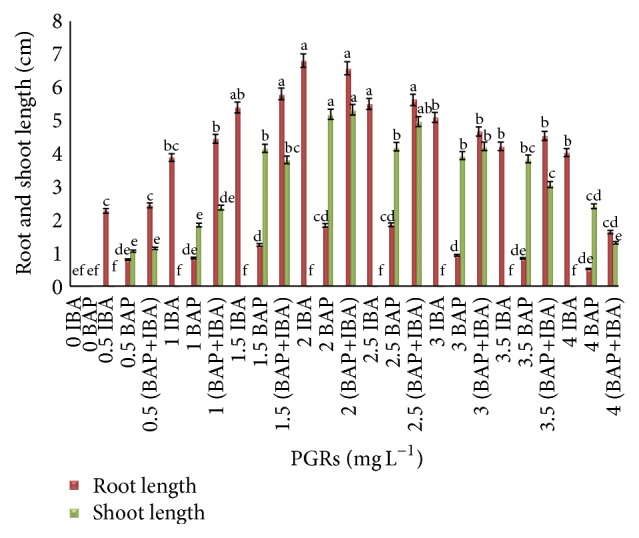
Root and shoot length from leaf explants of* C. latifolia* Dryand cultured on MS media supplemented with BAP and IBA alone or in combination (IBA+BAP) at various concentrations after 8 weeks of culture (*ρ* < 0.05, *n* = 4). The columns and bars represent mean + SE. The different letters at the top of columns in the same color indicate significant differences based on Duncan's multiple range tests.

**Figure 4:**
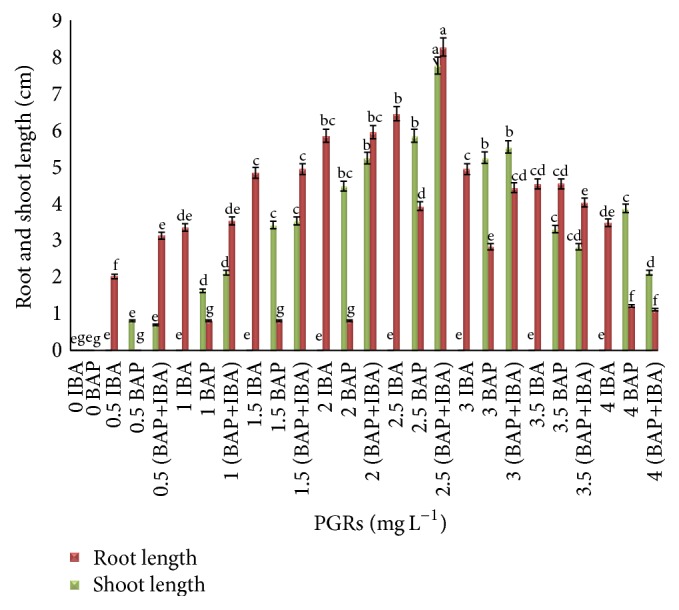
Root and shoot length from tuber explants of* C. latifolia* Dryand cultured on MS media supplemented with BAP and IBA alone or in combination (IBA+BAP) at various concentrations after 8 weeks of culture (*ρ* < 0.05, *n* = 4). The columns and bars represent mean + SE. The different letters at the top of columns in the same color indicate significant differences based on Duncan's multiple range tests.

The leaf explants cultured in MS media supplemented with combination of IBA and BAP (2 mg L^−1^) showed the optimum result of root and shoot length (6.61 ± 0.68 cm and 5.35 ± 1.31 cm), respectively (Figure 3). The lengths of roots and shoots from tuber explants were increased (8.2 ± 1.12 cm and 7.7 ± 0.28 cm) when the explants were cultured on the same media with a combination of 2.5 mg L^−1^ IBA and BAP (Figure 4).

**Figure 5:**
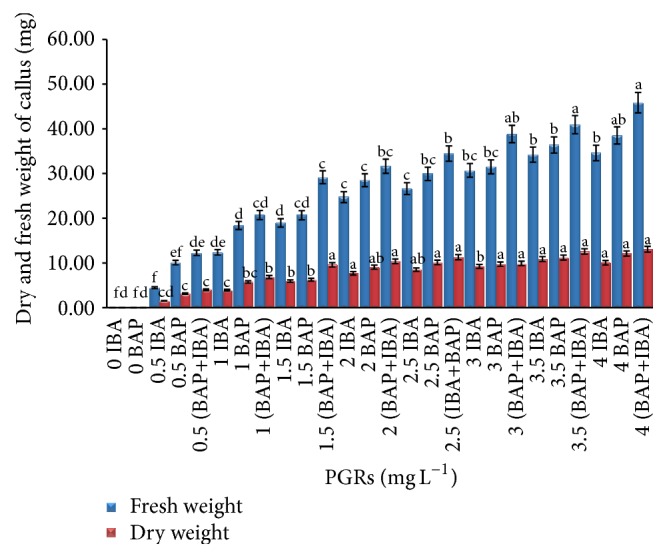
Fresh and dry matter yield from tuber explants of* C. latifolia* Dryand on MS media supplemented with BAP and IBA alone or in combination (IBA+BAP) at various concentrations after 8 weeks of culture. The columns and bars represent mean + SE. The different letters at the top of columns in the same color indicate significant differences based on Duncan's multiple range tests.

**Figure 6:**
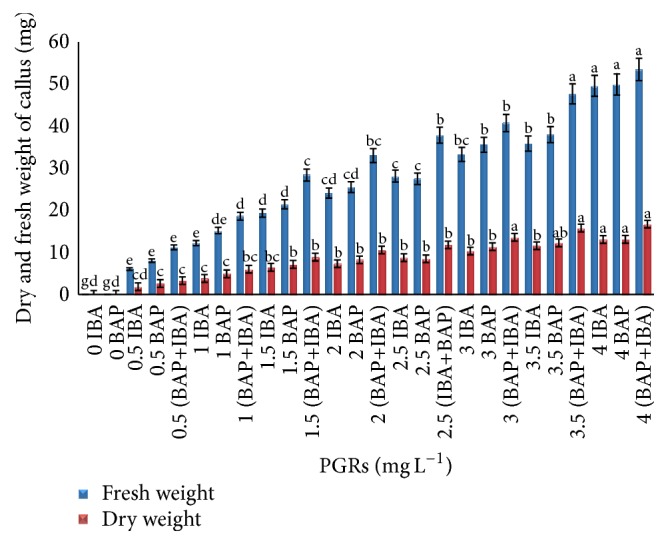
Fresh and dry matter yield from leaf explants of* C. latifolia* Dryand on MS media supplemented with BAP and IBA alone or in combination (IBA+BAP) at various concentrations after 8 weeks of culture. The columns and bars represent mean + SE. The different letters at the top of columns in the same color indicate significant differences based on Duncan's multiple range tests.

Based on the obtained results shown in Figure 5, tuber explants inoculated on MS media supplemented with combinations of 4 mg L^−1^ IBA and BAP showed the higher weight based on fresh (45.589 ± 1.45 g) and dry weight (12.805 ± 0.57 g). The leaf explants inoculated on the same media and PGRs showed higher fresh and dry weight (53.82 ± 1.45 g, 16.818 ± 0.87 g), respectively (Figure 6).

### 7.5. Acclimatization

The regenerated plants were kept for six weeks in the rooting medium and transferred to MS medium free of PGRs for two weeks. The plantlets were maintained under normal room temperature for 7-8 days before transplantation under semicontrolled temperature (30 ± 2°C) in a chamber with 80% humidity. The plants were transferred to the open place and gradually were acclimated to outdoor condition. The survival rate was measured as 89%.

### 7.6. Antioxidant Properties

The antioxidant activity of plant extracts (callus from leaf and tuber, resp.) of* C. latifolia* was compared with leaves from* in vivo* plants, butylated hydroxyl toluene (BHT) and ascorbic acid or vitamin C (1 mg L^−1^) as a positive control.

**Figure 7:**
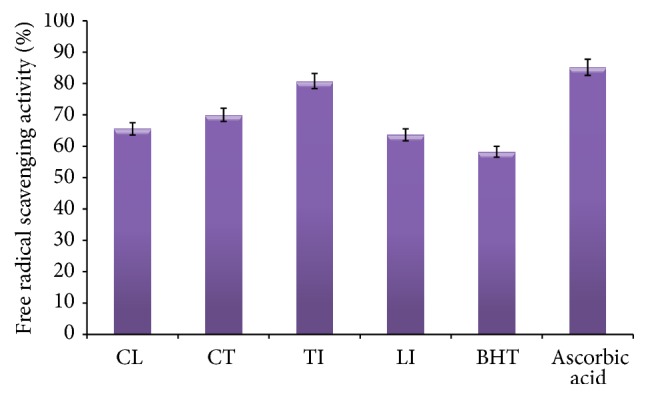
CL: callus from leaf, CT: callus from tuber, TI: tuber from intact plant (*in vivo*), and LI: leaf from intact plant (*in vivo*). Antioxidant activity of examining plant extracts (leaves from* in vivo* plants compared to callus from leaf and tuber, resp.) of* C. latifolia* Dryand (10 g L^−1^) measured using a DPPH^*∗*^ scavenging activity assay presented as a percentage value. Ascorbic acid and BHT (butylated hydroxyl toluene 1 mg L^−1^) were applied as the positive controls. All the values are average of triplicates. The data were analyzed by one-way ANOVA and the DPPH^*∗*^ scavenging activity percentage means of samples were compared using Duncan's Multiple comparison test *p* < 0.05 (DMCT).

**Figure 8:**
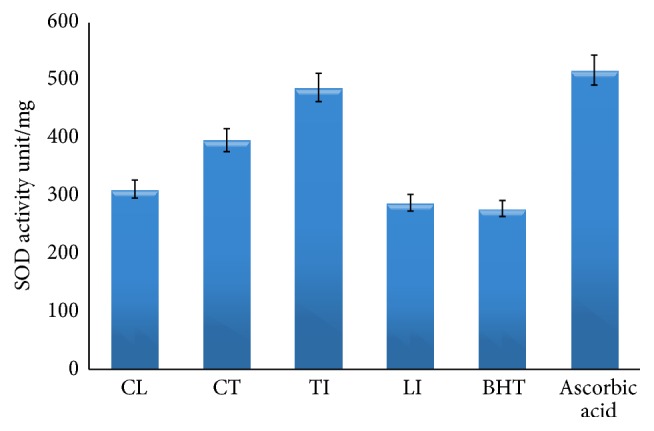
CL: callus from leaf, CT: callus from tuber, TI: tuber from intact plant (*in vivo*), and LI: leaf from intact plant (*in vivo*). Superoxide dismutase (SOD) was applied according to the kit protocol to examine SOD activities in plant extracts (leaves from* in vivo* plants compared to callus from leaf and tuber, resp.) of* C. latifolia* Dryand (10 g L^−1^). Ascorbic acid and BHT (butylated hydroxyl toluene 1 mg L^−1^) were applied as the positive controls. All the values are average of triplicates. The data were analyzed by one-way ANOVA and the results of samples were compared using Duncan's Multiple comparison test *p* < 0.05 (DMCT).

Based on the results in Figure 7, the free radical scavenging potential of callus from tuber extracts (70%) was higher than callus from leaf extracts (65%). In addition,* in vivo* tuber and leaf extract showed 80% and 60%, respectively. The same pattern was observed when SOD activity was applied (Figure 8).

### 7.7. Antibacterial Activities

The value of various* C. latifolia* extracts was investigated, quantitatively, by measuring the diameter of the inhibition zones around the discs.

The various extract of* C. latifolia* exhibited considerable antibacterial activity against both gram-negative and gram-positive bacteria at a concentration of 10 g L^−1^ (Table 3). However, tuber and leaf extracts from the intact plant (*in vivo*) and callus (*in vitro*) showed higher inhibition zone compared to other extracts on gram-negative bacteria, especially against Klebsiella spp. and* P. aeruginosa.*

## 8. Discussion

It is well known that* in vitro* establishment and regeneration of plants are influenced by various factors, such as explants type, the physiological status of* in vivo* plants, genotype, species, and media composition, type and combination of plant growth regulators, and culturing conditions.

In this study, among the different types of explants such as leaf, tuber, and seed,* in vitro* regeneration was only obtained from leaf and tuber explants. The explants cultured in MS medium in the absence of PGRs have showed no response to shoot and root induction. The response of tuber explants to* in vitro *regeneration in Hypoxidaceae species varied considerably.

Vinesi et al. [26] reported successful rhizome culture of* H. obtusa* (Hypoxidaceae) on MS medium supplemented with (1 mg L^−1^) BAP. Based on Page and Van Staden [27], more than 70% of shoot and root from corn explants of* H. rooperi* (Hypoxidaceae) were formed on MS media added with (1 mg L^−1^) BAP between six and ten weeks, respectively.

However, the best in multiplication response of* H. colchicifolia* reported on MS medium containing 2 mg L^−1^ BAP compared to other PGRs [28].

Appleton and van Staden [29] and Nsibande [30] reported regeneration in Hypoxidaceae family is varied with respect to the growth regulators requirement, shoot multiplication, and callus production. The leaf explants inoculated on MS media supplemented with various concentrations of BAP showed only a small amount of shoot formation in the absence of IBA. However, the shoot and root were induced when explants inoculated on MS medium with a combination of IBA and BAP. Nsibande [30] reported similar results on this family.

Based on our research, the antioxidant activities of this plant from leaves and tubers have not been reported. Even then, slight differences in the antioxidant activities do occur that solely depend on varieties, location, and growth conditions. Overall, in the estimation of the antioxidant capacities and the free radical scavenging assays showed positive results. The percent of inhibition in the DPPH assay and SOD activity were found more from tuber and leaf extraction (*in vivo*). According to Farzinebrahimi et al. [12], Farzinebrahimi et al. [13], and Khorasani et al. [14] different parts of the plants produced different antioxidant compounds or different amount of compounds possibly due to their degree of differences in gene expression. The ethanolic extracts of* H. hemerocallidea* from this family showed antioxidant properties via hydroxyl scavenging ability [10].* In vitro* studies indicated good ability to scavenge free radicals (hydroxyl ions) [31]. The antibacterial activity of the tuber may be due to presence of phenolic active compounds in [32]* C. latifolia*. Antibacterial effect against gram-negative and gram-positive bacteria could be as natural source for producing pharmacological products. The results of the current study supported the traditional treatment by medicinal plants and proposed antibacterial agents from plant extracts with antibacterial properties.

The maximum activity was observed against gram-negative and gram-positive bacteria for* C. latifolia* tuber extracts* in vivo* and* in vitro* as compared with leaf extract, respectively.

Antimicrobial properties of medicinal plants are being increasingly stated from various parts of the world. Based on The World Health Organization report, the plant active constituents are used as folk medicine in traditional therapies of 80% of the world's population. In this study, the tuber extracts obtained from* C. latifolia* (*in vivo* and* in vitro*) showed strong activity against most of the tested bacterial strains. The results were compared with standard antibiotic drug.

The effect of antibacterial in medicinal plants varies intensely depending on the phytochemical features of plant families and subfamilies and even the grown area [33, 34]. Our results revealed that the tuber of* Curculigo latifolia* Dryand has the most effective antibiotics against all the studied bacteria compared with other explants.

## 9. Conclusion

Combinations of BAP and IBA in MS media exhibited the highest average numbers of root and shoot formation from leaf and tuber explants. Our data revealed that frequent subculture was effective in reducing phenolic exudation; also, pretreatment of tubers with citric acid could eliminate browning during culture period. This study also showed that tuber extracts from* in vivo* gave higher results for antioxidant activity and inhibition zone against gram-negative bacteria compared to the same concentration of callus extract from tuber and leaf.

Further and more specific studies,* in vivo* or* in vitro*, are recommended to determine the characteristics of this species.

## Figures and Tables

**Table 1:** Mean number of shoots and roots' formation from tuber explants of *C. latifolia* Dryand cultured on MS media supplemented with BAP alone or in combination with IBA (IBA+BAP) at various concentrations after 8 weeks of culture (*ρ* < 0.05, *n* = 4). Different letters in the same column represent a significant difference at the 5% level in Duncan's multiple range tests.

PGRs	Mean number of shoots per leaf explant ± SE	Mean number of roots per leaf explant ± SE
Control	0^f^	0^g^
0.5 IBA	0^f^	2.3 ± 0.6^ef^
0.5 BAP	1 ± 0.2^e^	0^g^
0.5 (BAP+IBA)	1.3 ± 0.4^e^	4.3 ± 0.2^de^
1 IBA	0^f^	4.4 ± 0.4^de^
1 BAP	2.4 ± 0.8^d^	0.9 ± 0.2^f^
1 (BAP+IBA)	3.6 ± 0.6^d^	4.6 ± 0.9^de^
1.5 IBA	0^f^	5.5 ± 0.3^d^
1.5 BAP	4.4 ± 0.6^c^	0.9 ± 0.2^f^
1.5 (BAP+IBA)	4.5 ± 0.9^c^	5.9 ± 0.7^bc^
2 IBA	0	6.7 ± 0.1^ab^
2 BAP	5.4 ± 0.1^b^	0.9 ± 0.5^f^
2 (BAP+IBA)	6.8 ± 0.5^a^	6.9 ± 0.3^a^
2.5 IBA	0^f^	7.6 ± 0.5^a^
2.5 BAP	6.9 ± 0.3^a^	4.2 ± 0.8^de^
2.5 (BAP+IBA)	8.8 ± 1.0^a^	7.4 ± 1.0^a^
3 IBA	0^f^	5.4 ± 0.5^d^
3 BAP	6.8 ± 0.5^a^	3.2 ± 0.4^ef^
3 (BAP+IBA)	6.3 ± 0.1^ab^	5.5 ± 0.1^d^
3.5 IBA	0^f^	5.6 ± 0.8^d^
3.5 BAP	4.3 ± 0.4^c^	5.8 ± 0.6^cd^
3.5 (BAP+IBA)	3.5 ± 0.3^d^	5.1 ± 0.2^d^
4 IBA	0^f^	4.6 ± 0.4^de^
4 BAP	4.6 ± 0.3^c^	2.4 ± 0.2^ef^
4 (BAP+IBA)	3.4 ± 0.9^d^	1.3 ± 0.4^f^

**Table 2:** Mean number of shoots and roots' formation from leaf explants of *C. latifolia* Dryand cultured on MS media supplemented with BAP alone or in combination with IBA (IBA+BAP) at various concentrations after 8 weeks of culture (*ρ* < 0.05, *n* = 4). Different letters in the same column represent a significant difference at the 5% level in Duncan's multiple range tests.

PGRs	Mean number of shoots per leaf explant ± SE	Mean number of roots per leaf explant ± SE
Control	0^f^	0^e^
0.5 IBA	0^f^	3.3 ± 0.9^d^
0.5 BAP	1.4 ± 0.1^e^	0.9 ± 0.4^e^
0.5 (BAP+IBA)	1.0 ± 0.2^ef^	3.5 ± 0.5^d^
1 IBA	0^f^	4.9 ± 1.0^c^
1 BAP	1.8 ± 0.1^de^	1.0 ± 0.1^e^
1 (BAP+IBA)	2.4 ± 0.4^d^	5.8 ± 0.5^b^
1.5 IBA	0^f^	6.1 ± 0.4^b^
1.5 BAP	3.09 ± 0.3^bc^	1.4 ± 0.4^de^
1.5 (BAP+IBA)	4.4 ± 0.2^b^	6.7 ± 0.8^a^
2 IBA	0^f^	7.3 ± 0.1^a^
2 BAP	5.5 ± 0.4^a^	2.1 ± 0.1^d^
2 (BAP+IBA)	5.7 ± 0.8^a^	7.1 ± 0.5^a^
2.5 IBA	0^f^	6.6 ± 0.4^ab^
2.5 BAP	4.3 ± 0.6^b^	2.1 ± 0.8^d^
2.5 (BAP+IBA)	4.8 ± 0.5^ba^	6.8 ± 0.2^ab^
3 IBA	0^f^	6.1 ± 0.5^b^
3 BAP	3.9 ± 0.4^bc^	1.1 ± 0.4^e^
3 (BAP+IBA)	4.8 ± 0.7^ba^	5.8 ± 0.6^b^
3.5 IBA	0^f^	4.3 ± 0.8^bc^
3.5 BAP	3.7 ± 0.3^cb^	1.0 ± 0.7^e^
3.5 (BAP+IBA)	3.2 ± 0.2^c^	5.6 ± 1.0^b^
4 IBA	0^f^	5.1 ± 0.2^c^
4 BAP	2.4 ± 0.3^cd^	0.8 ± 0.5^e^
4 (BAP+IBA)	1.3 ± 0.1^de^	1.8 ± 0.3^d^

**Table 3:** Inhibition effect of 10 g L^−1^ of *C.latifolia* ethanoic extracts (leaf and tuber *in vitro* and *in vivo* generated) against the growth of four pathogenic bacteria.

	Tested microorganisms	Types of plants	Inhibitory zone (mm) ± standard deviation	Ampicillin (30 *μ*g) ± standard deviation
Gram-positive bacteria	*S. aureus*	Leaf extracts * in vitro*	*7.1* ± 1.54^d^	18 ± 2.84^a^
		Tuber extract *in vitro*	*12.1* ± 1.14^b^	18 ± 2.57^a^
		Tuber extract *in vivo*	*13.8* ± 1.24^b^	18.2 ± 1.14^a^
		Leaf extracts * in vivo*	*7.8* ± 1.47^c^	18.7 ± 2.64^a^
	*Bacillus cereus*	Leaf extracts * in vitro*	7.0 ± 1.30^e^	25 ± 3.60^a^
		Tuber extract *in vitro*	12.5 ± 1.10^c^	25 ± 3.10^a^
		Tuber extract *in vivo*	*17.3* ± 1.24^b^	25 ± 3.10^a^
		Leaf extracts * in vivo*	7.2 ± 1.10^d^	25 ± 1.60^a^

Gram-negative bacteria	*Klebsiella *sp.	Leaf extracts * in vitro*	7.1 ± 1.30^d^	24 ± 3.20^a^
		Tuber extract *in vitro*	17.4 ± 1.10^b^	24 ± 4.30^a^
		Tuber extract *in vivo*	*22.3 * ± 1.24^ab^	24 ± 3.20^a^
		Leaf extracts * in vivo*	7.4 ± 1.10^c^	24 ± 3.20^a^
	*P. aeruginosa*	Leaf extracts * in vitro*	*10.2* ± 1.50^c^	31 ± 2.10^a^
		Tuber extract *in vitro*	*18.8* ± 1.7^b^	30.1 ± 1.80^a^
		Tuber extract *in vivo*	*25.4* ± 1.01^ab^	30.1 ± 1.80^a^
		Leaf extracts * in vivo*	*10.7* ± 1.10^bc^	30.2 ± 1.10^a^

Inhibition zone in mm (5 mm diameter of disk) as the means of triplicate of experiments. The data were analysed by one-way ANOVA and the inhibition means of samples were compared using Duncan's multiple comparison test (DMCT). Different letters in the same column represent a significant difference at the 5% level in Duncan's multiple range tests.

## References

[1] Kocyan A. (2007). The discovery of polyandry in Curculigo (Hypoxidaceae): implications for androecium evolution of asparagoid monocotyledons. *Annals of Botany*.

[2] Ismail M. F., Psyquay Abdulla N., Saleh G. B., Ismail M. (2010). Anthesis and flower visitors in *Curculigo latifolia* dryand. *Journal of Biology and Life Science*.

[3] Farzinebrahimi R., Taha R. M., Rashid K. A. (2013). Effect of light intensity and soil media on establishment and growth of *Curculigo latifolia* Dryand. *Journal of Applied Horticulture*.

[4] Shaari N. Lemba (*Curculigo latifolia*) leaf as a new materials for textiles.

[5] Ahmad F. B., Holdsworth D. K. (1994). Medicinal plants of Sabah, Malaysia, part II. The muruts. *International Journal of Pharmacognosy*.

[6] Wiart C. (2000). *Medicinal Plant of Southeast Asia*.

[7] Yamashita H., Theerasilp S., Aiuchi T., Nakaya K., Nakamura Y., Kurihara Y. (1990). Purification and complete amino acid sequence of a new type of sweet protein with taste-modifying activity, curculin. *The Journal of Biological Chemistry*.

[8] Kant R. (2005). Sweet proteins-potential replacement for artificial low calorie sweeteners. *Nutrition Journal*.

[9] Masuda T., Kitabatake N. (2006). Developments in biotechnological production of sweet proteins. *Journal of Bioscience and Bioengineering*.

[10] Nie Y., Dong X., He Y. (2013). Medicinal plants of genus *Curculigo*: traditional uses and a phytochemical and ethnopharmacological review. *Journal of Ethnopharmacology*.

[11] Grzegorczyk I., Matkowski A., Wysokińska H. (2007). Antioxidant activity of extracts from *in vitro* cultures of *Salvia officinalis* L.. *Food Chemistry*.

[12] Farzinebrahimi R., Taha R. M., Rashid K., Yaacob J. S. (2014). The effect of various media and hormones via suspension culture on secondary metabolic activities of (Cape Jasmine) *Gardenia jasminoides* ellis. *The Scientific World Journal*.

[13] Farzinebrahimi R., Taha R. M., Fadaie Nasab M. (2012). *In vitro* plant regeneration, antioxidant and antibacterial studies on broccoli, *Brassica oleracea* var. italica. *Pakistan Journal of Botany*.

[14] Khorasani A., Sani W., Philip K., Taha R. M., Rafat A. (2010). Antioxidant and antibacterial activities of ethanolic extracts of *Asparagus officinalis* cv. Mary Washington: Comparison of *in vivo* and *in vitro* grown plant bioactivities. *African Journal of Biotechnology*.

[15] Rafat A., Koshy P., Sekaran M. (2010). Antioxidant potential and content of phenolic compounds in ethanolic extracts of selected parts of *Andrographis paniculata*. *Journal Medicinal Plants Research*.

[16] Bakrudeen A., Subha Shanthi G., Gouthaman T., Kavitha M., Rao M. (2011). *In vitro* micropropagation of *Catharanthus roseus*—an anticancer medicinal plant. *Acta Botanica Hungarica*.

[17] Babaei N., Abdullah N. A. P., Saleh G., Abdullah T. L. (2014). An efficient *in vitro* plantlet regeneration from shoot tip cultures of *Curculigo latifolia*, a medicinal plant. *The Scientific World Journal*.

[18] Francis S. V., Senapati S. K., Rout G. R. (2007). Rapid clonal propagation of *Curculigo orchioides* Gaertn., an endangered medicinal plant. *In Vitro Cellular & Developmental Biology—Plant*.

[19] Thomas T. D. (2007). High-frequency, direct bulblet induction from rhizome explants of *Curculigo orchioides* Gaertn., an endangered medicinal herb. *In Vitro Cellular & Developmental Biology*.

[20] Wala B. B., Jasrai Y. T. (2003). Micropropagation of an endangered medicinal plant *Curculigo orchioides* Gaertn. *Plant Tissue Culture*.

[21] Lim-Ho C. L. (1981). *Tissue Culture of Curculigo latifolia Dry. ex W.T. Ait. (Hypoxidaceae)*.

[22] Taha R. M. (1993). Tissue culture studies of *Citrus hystrix* D.C. and *Severinia buxifolia* (poir) tenore. *Asia-Pacific Journal of Molecular Biology and Biotechnology*.

[23] Murashige T., Skoog F. (1962). A revised medium for rapid growth and bio assays with tobacco tissue cultures. *Physiologia Plantarum*.

[24] Ahmed A. B. A., Pallela R., Rao A. S., Rao M. V., Mat Taha R. (2011). Optimized conditions for callus induction, plant regeneration and alkaloids accumulation in stem and shoot tip explants of Phyla nodiflora. *Spanish Journal of Agricultural Research*.

[25] Gomez K. A., Gomez A. A. (1976). *Statistical Procedures for Agricultural Research with Emphasis on Rice*.

[26] Vinesi P., Serafini M., Nicoletti M., Spanò L., Betto P. (1990). Plant regeneration and hypoxoside content in *Hypoxis obtusa*. *Journal of Natural Products*.

[27] Page Y. M., Van Staden J. (1984). *In vitro* propagation of *Hypoxis rooperi*. *Plant Cell, Tissue and Organ Culture*.

[28] Appleton M. R., Ascough G. D., Van Staden J. (2012). *In vitro* regeneration of *Hypoxis colchicifolia* plantlets. *South African Journal of Botany*.

[29] Appleton M. R., van Staden J. (1995). Micropropagation of some South African *Hypoxis* species with medicinal and horticultural potential. *Acta Horticulturae*.

[30] Nsibande B. E. (2012). *In vitro regeneration of four hypoxis species and transformation of Camelina sativa and Crambe abyssinica [M.S. thesis]*.

[31] Mahomed I. M., Ojewole J. A. O. (2003). Hypoglycemic effect of *Hypoxis hemerocallidea* corm (*African potato*) aqueous extract in rats. *Methods and Findings in Experimental and Clinical Pharmacology*.

[32] Nagesh K. S., Shanthamma C. (2009). Antibacterial activity of *Curculigo orchioides* rhizome extract on pathogenic bacteria. *African Journal of Microbiology Research*.

[33] Al-Mariri A., Safi M. (2014). *In vitro* antibacterial activity of several plant extracts and oils against some gram-negative bacteria. *Iranian Journal of Medical Sciences*.

[34] Sarac N., Ugur A. (2009). The *in vitro* antimicrobial activities of the essential oils of some *Lamiaceae* species from Turkey. *Journal of Medicinal Food*.

